# Beyond kappa: an informational index for diagnostic agreement in dichotomous and multivalue ordered-categorical ratings

**DOI:** 10.1007/s11517-020-02261-2

**Published:** 2020-11-03

**Authors:** Alberto Casagrande, Francesco Fabris, Rossano Girometti

**Affiliations:** 1 Dipartimento di Matematica e Geoscienze, Università degli Studi di Trieste, Trieste, Italy; 2 Dipartimento di Area Medica, Istituto di Radiologia, Ospedale S. Maria della Misericordia, Università degli Studi di Udine, Udine, Italy

**Keywords:** Diagnostic agreement, Cohen’s kappa statistic, Multivalue ordered-categorical ratings, Inter-reader agreement, Information measures

## Abstract

Agreement measures are useful tools to both compare different evaluations of the same diagnostic outcomes and validate new rating systems or devices. Cohen’s kappa (*κ*) certainly is the most popular agreement method between two raters, and proved its effectiveness in the last sixty years. In spite of that, this method suffers from some alleged issues, which have been highlighted since the 1970s; moreover, its value is strongly dependent on the prevalence of the disease in the considered sample. This work introduces a new agreement index, the *informational agreement* (*IA*), which seems to avoid some of Cohen’s kappa’s flaws, and separates the contribution of the prevalence from the nucleus of agreement. These goals are achieved by modelling the agreement—in both dichotomous and multivalue ordered-categorical cases—as the information shared between two raters through the virtual *diagnostic channel* connecting them: the more information exchanged between the raters, the higher their agreement. In order to test its fair behaviour and the effectiveness of the method, *IA* has been tested on some cases known to be problematic for *κ*, in the machine learning context and in a clinical scenario to compare *ultrasound* (US) and *automated breast volume scanner* (ABVS) in the setting of breast cancer imaging.

**Graphical Abstract:**
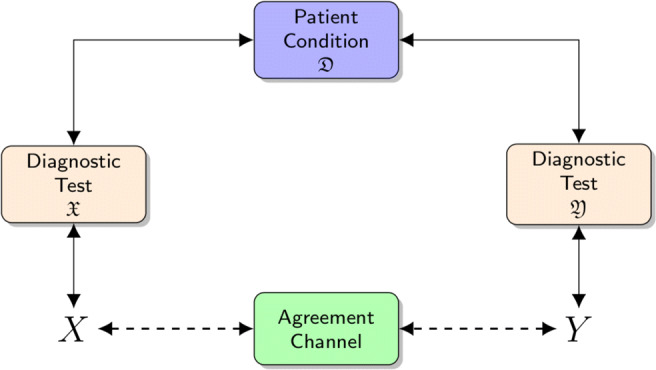
To evaluate the agreement between the two raters $\mathfrak {X}$ and $\mathfrak {Y}$ we create an *agreement channel*, based on Shannon Information Theory, that directly connects the random variables *X* and *Y*, that express the raters outcomes. They are the terminals of the chain *X*⇔ diagnostic test performed by $\mathfrak {X}$ ⇔ patient condition$\mathfrak {D}$ ⇔ diagnostic test performed by $\mathfrak {Y}$ ⇔ *Y*

To evaluate the agreement between the two raters $\mathfrak {X}$ and $\mathfrak {Y}$ we create an *agreement channel*, based on Shannon Information Theory, that directly connects the random variables *X* and *Y*, that express the raters outcomes. They are the terminals of the chain *X*⇔ diagnostic test performed by $\mathfrak {X}$ ⇔ patient condition$\mathfrak {D}$ ⇔ diagnostic test performed by $\mathfrak {Y}$ ⇔ *Y*

## Introduction

Diagnostic agreement is a measure to both appraise the reliability of a diagnostic exam and evaluate the accordance between different interpretations of the same diagnostic results. The very same approach has successfully been used also in different domains, such as machine learning, to identify noise in data sets and to compare multiple predictors in ensemble methods (e.g. see [40, 45]). Many different techniques have been introduced so far to gauge diagnostic agreement. For instance, raw agreement [2], Cohen’s kappa [13], intraclass correlation [44], McNemar’s test [34], and log odds ratio [22] have been proposed for the dichotomous analysis, i.e. when the scale accounts only two admissible values; on the contrary, weighted kappa [14], Fleiss-Cohen (quadratic) weights [23], intraclass correlation [2, 44], and association models [7] have been proposed for multivalue ordered-categorical ratings, i.e. when the admissible values are more than 2. Even though Cohen’s kappa suffers from a set of long-debated inconsistencies between its value and the expectations of clinicians [6, 12, 15, 16, 20, 26, 43, 49–51, 53], it is de facto the standard technique for diagnostic agreement, and it is used in the vast majority of real case analysis.

Recently, the relation between patient condition and the corresponding rater evaluation has been modelled as a virtual *diagnostic information channel* that transmits the exam outcomes [24]: the assumptions of a rater $\mathfrak {X}$ are based on the information obtained by the diagnostic channel, and figure out the patient condition $\mathfrak {D}$ (see Fig. 1 in [24]): the more information on the patient status flows from the patient’s real condition to the rater evaluations though the diagnostic information channel, the more accurate the diagnostic test. The channel might represent a mammography to be interpreted by a radiologist, or a *prostate-specific antigen* (PSA) level measure, aiding the urologist to decide whether the cut-off has been exceeded. In this context, a coherent measure of the quality of a diagnostic test is the amount of information that can be extracted from the diagnostic channel, which is the *mutual information* (*MI*) introduced in *Shannon’s information theory* (*IT*) to evaluate the flow of information exchanged between two random variables [41].

**Fig. 1:**
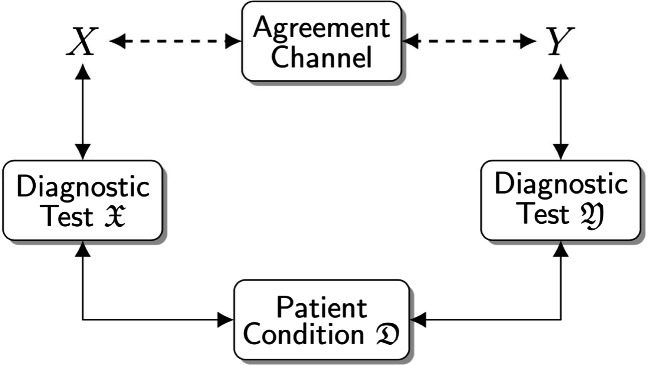
The *agreement channel* directly connects the random variables *X* and *Y* that are the terminals of the chain *X*⇔ diagnostic test performed by $\mathfrak {X}$ ⇔ patient condition $\mathfrak {D}$ ⇔ diagnostic test performed by $\mathfrak {Y}$ ⇔ *Y*

While *IT* tools have widely been suggested in medical statistics and diagnostics (e.g. see [4, 5, 8–10, 35–37, 46, 47]), they have not been used as broadly in relation with agreement measures. On this research track, we can only mention [31], where a normalised weighted *MI* is used as an index of *intercoder agreement*, and [29], which uses *MI* to quantify the information shared between outcomes of multiple healthcare surveys.

This work focuses on measuring the diagnostic agreement between two raters, $\mathfrak {X}$ and $\mathfrak {Y}$, by using the *IT* approach on the corresponding diagnostic channels. If *X* and *Y* are the random variables representing the rater evaluations, then the more information is virtually exchanged between *X* and *Y* throughout the *agreement channel*—consisting of the concatenation of two diagnostic channels—the greater is the agreement between the two raters (see Fig. 1). The proposed approach is adherent to Shannon’s vision of a communication channel, carrying in this case diagnostic information, as opposed as those presented in [29, 31], which introduce agreement measures inspired by *MI*, but lose a direct connection with *IT*. In this sense, our approach is new in the agreement domain.

The aim of this work is manifold; it (i) presents an *informational agreement* index (*IA*) *à la Shannon*, for both the dichotomous and the multivalue ordered-categorical cases, (ii) shows that *IA* conceptually generalises Cohen’s kappa, (iii) proves that *IA* corrects some of the flaws of Cohen’s kappa, and, finally, (iv) justifies the use of our approach in real cases, by applying it to a medical data set coming from the literature.

## Methods

### Basic notions

We consider a generic multivalue ordered-categorical scale having *q* levels (ratings), i.e. [1,2,…,*q*]; when *q* = 2 we fall back to the standard dichotomous case, while, for instance, the classical 5-point malignancy scale for breast cancer can be modelled by imposing *q* = 5.

The patient condition $\mathfrak {D}$ is a hidden status, and raters $\mathfrak {X}$ and $\mathfrak {Y}$ make assumptions about it by means of a likelihood expressed in the *q*-level scale of ratings. The random variables *X* and *Y* are associated with the rates of $\mathfrak {X}$ and $\mathfrak {Y}$, respectively. Both raters perform *N* evaluations of the same data set. We write *n*_*X*_(*z*) (*n*_*Y*_(*z*)) to denote the number of evaluations attributed to $\mathfrak {X}$ ($\mathfrak {Y}$), whose rate is *z*. Since ${\sum }_{x} n_{X}(x) = N$, the probability *p*_*X*_(*x*) for *X* to equals *x* is *n*_*X*_(*x*)/*N*; analogously, ${\sum }_{y} n_{Y}(y) = N$ and the probability *p*_*Y*_(*y*) for *Y* to equals *y* is *n*_*Y*_(*y*)/*N*. $P_{X} = \left \{{p}_{X}({x}) \right \}_{x} $ and $P_{Y} = \left \{{p}_{Y}({y}) \right \}_{y} $ are the probability distributions (p.d.) for ratings of $\mathfrak {X}$ and $\mathfrak {Y}$, respectively.

Depending on the goal of the analysis, $\mathfrak {X}$ and $\mathfrak {Y}$ may represent either two raters having dissimilar experiences, the same rater using different diagnostic tests, or the same rater repeating the same test in distinct moments. In all these cases, discrepancies between $\mathfrak {X}$ and $\mathfrak {Y}$ are possible and, in non-trivial situations, expected. The number of samples which are rated *x* by $\mathfrak {X}$ and, at the same time, *y* by $\mathfrak {Y}$, is denoted by *n*(*x*,*y*). Since ${\sum }_{x y} n(x,y) = N$, the joint probability, *p*_*XY*_(*x*,*y*), for a sample to be rated *x* by $\mathfrak {X}$ and *y* by $\mathfrak {Y}$ is *n*(*x*,*y*)/*N*. The joint probability distribution for *X* and *Y* is *P*_*XY*_ = {*p*_*XY*_(*x*,*y*)}_*x*,*y*_. It is easy to see that ${\sum }_{y} {p}_{XY}({x},{y})={p}_{X}({x})$, ${\sum }_{x} {p}_{XY}({x},{y})={p}_{Y}({y})$ and ${\sum }_{x y} {p}_{XY}({x},{y})=1$ hold.

By Bayes’ theorem, the conditional probability $P_{Y/X}=\left \{{p}_{Y/X}({y}/{x})\right \}_{x,y}$—i.e. the probability to obtain a rate *y* by $\mathfrak {Y}$ knowing that *x* is the rate of $\mathfrak {X}$—is equal to *p*_*XY*_(*x*,*y*)/*p*_*X*_(*x*). The conditional probabilities *p*_*Y*/*X*_(*y*/*x*), for all *x*,*y* ∈ [1,*q*], constitute the entries of the *channel transition matrix* *Γ*_*q*_

1 $$ {{\varGamma}}_{q} \overset{\text{def}}{=} \left( \begin{array}{llll} {p}_{Y/X}({1}/{1}) & {p}_{Y/X}({1}/{2}) & {\cdots} & {p}_{Y/X}({1}/{q}) \\ {p}_{Y/X}({2}/{1}) & {p}_{Y/X}({2}/{2}) & {\cdots} & {p}_{Y/X}({2}/{q}) \\ \quad \quad {\vdots} & \quad\quad {\vdots} & \ \ {\vdots} & \quad \quad{\vdots} \\ {p}_{Y/X}({q}/{1}) & {p}_{Y/X}({q}/{2}) & {\cdots} & {p}_{Y/X}({q}/{q}) \end{array} \right)  $$

introduced by Shannon [41].

Note that when $\mathfrak {Y}$ is a binary rater that tests the presence/absence of a condition $\mathfrak {X}$, the *sensitivity* and *specificity* of $\mathfrak {Y}$ are the probabilities, for a rate of $\mathfrak {Y}$, to be correct given the presence and the absence of $\mathfrak {X}$, respectively. Thus, if the stochastic variable *X* associated to $\mathfrak {X}$ is such that *X* = 1 if and only if $\mathfrak {X}$ is present, then the sensitivity and the specificity of $\mathfrak {Y}$ are *p*_*Y*/*X*_(1/1) = *Γ*_2_(1,1) and *p*_*Y*/*X*_(2/2) = *Γ*_2_(2,2), respectively.

The *Shannon entropy* *H*(*X*) of a random variable *X* [41] is formally defined as

2 $$ H(X) \overset{\text{def}}{=} - \underset{x \in \mathcal{X}}{\sum} {p}_{X}({x}) \log_{q} {p}_{X}({x})  $$

where $\mathcal {X}$ is the set of the possible values for *X* and $q=|\mathcal {X}|$. This function measures the quantity of information carried by the variable *X* and is upper bounded by $\log _{q} (|\mathcal {X}|)$. Note that the Shannon entropy is not just one of the possible approaches attaining this goal, but it is *the only one* that satisfies some basic postulates required to coherently define an information measure [1, 30].

While the entropy *H*(*Y* ) gauges the quantity of information in *Y* by assuming no prior knowledge on it, in some cases a partial insight of *X* itself is already available. The conditional entropy of *Y* given *X* [41] is defined as

3 $$ H(Y/X) \overset{\text{def}}{=} -\underset{x \in \mathcal{X},y \in \mathcal{Y}}{\sum}{p}_{XY}({x},{y}) \log_{q} p_{Y/X}(y/x)  $$

and quantifies the information brought by the random variable *Y* assuming that the value of *X* is already known. Of course, if *Y* and *X* are completely independent, then *H*(*Y*/*X*) equals *H*(*Y* ); otherwise we have *H*(*Y*/*X*) < *H*(*Y* ).

The mutual information *MI*(*X*,*Y* ) [41] measures the stochastic (oriented) “distance” between the joint p.d. *P*_*XY*_ and the product of the marginals *P*_*X*_*P*_*Y*_; in other words it measures the stochastic dependence between two random variables *X* and *Y*: the greater *MI*(*X*,*Y* ), the more information exchanged between the variables [32] and, in our settings, between the raters $\mathfrak {X}$ and $\mathfrak {Y}$. *MI*(*X*,*Y* ) is formally defined as:

4 $$ {\textit{MI}}(X,Y) \overset{\text{def}}{=} \underset{x \in \mathcal{X},y \in \mathcal{Y}}{\sum} {p}_{XY}({x},{y})\log_{q}\frac{{p}_{XY}({x},{y})}{{p}_{X}({x}){p}_{Y}({y})}. $$

*MI* is symmetric, i.e. *MI*(*X*,*Y* ) = *MI*(*Y*,*X*), and it is easy to prove that

5 $$ {\textit{MI}}(X,Y) = H(Y)-H(Y/X) = H(X)-H(X/Y) \geq 0  $$

### Cohen’s kappa

Cohen’s kappa (*κ*) was introduced in [13] to measure the agreement between two raters, $\mathfrak {X}$ and $\mathfrak {Y}$. The idea is that of trying to gauge the “distance” of the joint probability distribution *P*_*XY*_ from the probability distribution *P*_*X*_*P*_*Y*_ (the product of the marginals), which models the independence between *X* and *Y*. In the dichotomous case, i.e. *q* = 2, these distributions can be represented by the following two matrices:

$$ O_{XY} \overset{\text{def}}{=} \left( \begin{array}{ll} {p}_{XY}({1},{1}) & {p}_{XY}({1},{2}) \\ {p}_{XY}({2},{1}) & {p}_{XY}({2},{2}) \end{array} \right) E_{XY} \overset{\text{def}}{=} \left( \begin{array}{ll} {p}_{X}({1}){p}_{Y}({1}) & {p}_{X}({1}){p}_{Y}({2}) \\ {p}_{X}({2}){p}_{Y}({1}) & {p}_{X}({2}){p}_{Y}({2}) \end{array} \right) $$

The *observed agreement*, *p*_*o*_, is defined as the global probability of a match between raters’ evaluations and can be computed as the sum of the elements in the main diagonal of *O*_*XY*_, i.e. *p*_*o*_ = *p*_*XY*_(1,1) + *p*_*XY*_(2,2). In opposition, the *expected agreement*, *p*_*e*_, is the global probability of a match explained by chance—so, assuming there is no correlation between the evaluations of the two raters—and corresponds to the sum of the elements in the main diagonal of *E*_*XY*_, i.e. *p*_*e*_ = *p*_*X*_(1)*p*_*Y*_(1) + *p*_*X*_(2)*p*_*Y*_(2). Cohen’s kappa can be defined on the basis of these two estimators as follows:

6 $$  \kappa \overset{\text{def}}{=} \frac{p_{o} - p_{e}}{1-p_{e}} $$

where the numerator is the observer agreement (*p*_*o*_) reduced by the probability that agreements are due to chance (*p*_*e*_), and the denominator is only meant to normalise the value in the interval [− 1,1].

From the theoretical point of view, *κ* has two main deficiencies. First of all it does not model the gain of information due to the diagnostic test, which is made in order to gain information on the disease. Second, and foremost, *κ* only considers the elements in the main diagonals of both *E*_*XY*_ and *O*_*XY*_; the remaining part of these matrices are, in some sense, related to the disagreement between variables, and all the negative values are flattened to the lack of an agreement.

There are cases in which the value of *κ* does not match the expectations of clinicians; some of these are described as alleged pitfalls in literature, while others seem to be structural flaws due to the way in which the agreement is evaluated.

It was proven in [11, 43] that the value of *κ* is affected by the *prevalence of the condition*, i.e. the probability for the condition $\mathfrak {D}$ to be present on a subject. Feinstein and Cicchetti [20] described two situations that lead to alleged paradoxes. In the first one, a relatively low value of *κ* can be obtained even if the subjects on which the two classification methods agree are much more than those in which they do not—i.e. *n*(1,1) + *n*(2,2) ≫ *n*(1,2) + *n*(2,1) −. This situation occurs when the marginal totals are *highly symmetrically unbalanced* (SU), that is when either *n*_1._ ≫ *n*_2._ (*n*_1._ ≪ *n*_2._), or *n*_.1_ ≫ *n*_.2_ (*n*_.1_ ≪ *n*_.2_), where $n_{i.} \overset {\text {def}}{=} {\sum }_{j \in [1,q]} n(i,j)$ and $n_{.j} \overset {\text {def}}{=} {\sum }_{i \in [1,q]} n(i,j)$. In the second scenario, unbalanced marginal totals produce *κ* values greater than those due to more balanced totals. This case, known as *asymmetrical unbalanced marginals* (AU), happens when *n*_1._ > *n*_2._, while *n*_.1_ < *n*_.2_, or vice versa.

In Section 2.3, we propose an information theoretical agreement index, alternative to *κ*, which aims at mitigating the issues discussed above.

### Modelling diagnostic agreement by information theory

In our setting, we measure the agreement between two raters by modelling it as the quantity of information flowing through the *agreement channel* (*AC*), which is a virtual channel connecting the random variables *X* and *Y* by using the information path *X* ⇒ rating by $\mathfrak {X}$ ⇒ condition $\mathfrak {D}$⇒ rating by $\mathfrak {Y}$ ⇒ *Y* (see Fig. 1).

Since *MI*(*X*,*Y* ) is a measure of the stochastic dependence between *X* and *Y*, one might think of using it in order to gauge the agreement between $\mathfrak {X}$ and $\mathfrak {Y}$. Note, from Eq. (5), that the entropy, the conditional entropy, and the mutual information are strictly tied, and that

7 $$  \textit{MI}(X,Y) \leq \min \{H(X), H(Y)\} $$

Since the more uniform is the probability distribution of a random variable, the higher is the entropy of that variable [41], Eq. (7) means that the mutual information is upper bounded by the ineffectiveness of $\mathfrak {X}$ and $\mathfrak {Y}$ in distributing the sampled subjects into *q* classes—possibly, with no relation with the real conditions of the subjects having the same cardinality. For instance, if $\mathfrak {X}$ classifies almost all of the sampled subjects in the same way, being either “*having the condition*” or not, then both *H*(*X*) and *MI*(*X*,*Y* ) are about 0, even when $\mathfrak {X}$ and $\mathfrak {Y}$ are the same rater. This unwanted behaviour can be overcome by normalising *MI*(*X*,*Y* ) with respect to ${\min \limits } \{H(X), H(Y)\}$; this defines the *informational agreement* (*IA*)

8 $$ {{\textit{IA}}}({X},{Y}) \overset{\text{def}}{=} \frac{\textit{MI}(X,Y)}{\min \{H(X), H(Y)\}} $$

By using Eq. (7), it is easy to prove that *IA*’s value ranges in the interval [0,1]. So, the informational agreement retains all the information theoretic benefits of measuring the agreement by using the mutual information and, at the same time, mitigates the concerns about the dependency of *MI*(*X*,*Y* ) on the entropies of *X* and *Y*.

As opposed to *κ*, *IA* correctly measures the stochastic distance between *P*_*XY*_ and *P*_*X*_*P*_*Y*_, that is the distance of the two raters from the condition of independence; this is made by taking into account both the agreement and the disagreement components of the joint probability distribution of the rates. Moreover, it has a precise meaning from the informational point of view, because it represents the (normalised) amount of information exchanged between the two raters. In this sense, *IA* *is a natural completion of Cohen’s* *κ* in measuring the agreement.

### Prevalence of the condition and agreement indexes

By applying Bayes’ theorem to Eq. (4), we can deduce that

9 $$  \textit{MI}(X,Y)= \underset{x \in \mathcal{X},y \in \mathcal{Y}}{\sum} {p}_{X}({x}) {p}_{Y/X}({y}/{x})\log_{q} \frac{{p}_{Y/X}({y}/{x})}{ {\sum}_{z \in \mathcal{X}} {p}_{X}({z}){p}_{Y/X}({y}/{z})}. $$

Thus, *MI* is exclusively dependent on the elements *p*_*Y*/*X*_(*y*/*x*) of *Γ*_*q*_ and on the probability distribution *P*_*X*_.

Since, in our setting, the matrix *Γ*_*q*_ models the agreement channel, it *represents the relation between *$\mathfrak {X}$ *and *$\mathfrak {Y}$ *and is immutable with respect to the channel input*. Hence, *Γ*_*q*_ is not affected by the prevalence of the condition whose contribution is instead totally discharged on *P*_*X*_. While *MI* is still dependent on the prevalence, Eq. (9) conceptually insulates the essential nucleus of agreement, associated with the matrix *Γ*_*q*_, from the prevalence of the condition, which is embedded in *P*_*X*_. It is important to stress that we are not stating that *P*_*X*_ is fully determined by the prevalence; in fact, it also depends on the way in which $\mathfrak {X}$ partitions the sampled subjects.

Because of Eqs. (8) and (9), *IA* exclusively depends on *P*_*X*_, *Γ*_*q*_ and *H*(*Y* ), that is on *P*(*Y* ). But, since ${p}_{Y}({y})={\sum }_{x} {p}_{XY}({x},{y}) $ and *p*_*XY*_(*x*,*y*) = *p*_*Y*/*X*_(*y*/*x*)*p*_*X*_(*x*), *IA*, too, is fully determined by *Γ*_*q*_ and *P*_*X*_.

### *IA* validation

In order to validate *IA*, we compare it with *κ* by considering the six distinct dichotomous scenarios (i.e. *q* = 2) whose classification matrices are reported on Table 1. These scenarios were selected as representative of some *κ*’s flaws and, in particular, Table 1d and e are pinpointed in the literature as problematic for it. For each of them, we analysed the values of *IA* and *κ* together with the common sense expectations on the specific case, so as to highlight any possible inconsistency between them.

**Table 1:** The scenarios examined to compare *IA* and *κ*

	X			X		
	(a) Scenario 1			(b) Scenario 2		
Y	3600	2595	*6195*	9901	64	*9965*
	65	3740	*3805*	2	33	*35*
	*3665*	*6335*		*9903*	*97*	
	***κ*** **= 0.500**	***IA*** **= 0.309**		***κ*** **= 0.497**	***IA*** = **0.651**	
	(c) Scenario 3			(d) Scenario 4		
Y	9900	86	*9986*	21	5	*26*
	1	13	*14*	3	21	*24*
	*9901*	*99*		*24*	*26*	
	***κ*** **= 0.228**	***IA*** **= 0.541**		***κ*** **= 0.681**	***IA*** **= 0.371**	
	(e) Scenario 5			(f) Scenario 6		
Y	40	5	*45*	40	2	*42*
	3	2	*5*	3	5	*8*
	*43*	*7*		*43*	*7*	
	***κ*** **= 0.245**	***IA*** **= 0.073**		***κ*** **= 0.608**	***IA*** **= 0.342**	

*IA* and *κ* were also evaluated on the data published in [25] to compare conventional hand-held *ultrasound* (US) and *automated breast volume scanner* (ABVS) [42] in the setting of breast cancer imaging (see Tables 2 and 3).

**Table 2:** Raw agreement data between ultrasound (US) and automated breast volume scanner (ABVS) in assessing breast cancer findings according to all BI-RADS classes (***BR*** in the table for brevity)

		US					
		*BR* 1	*BR* 2	*BR* 3	*BR* 4	*BR* 5	Total
ABVS	*BR* 1	51	4	0	1	1	*57*
	*BR* 2	3	78	1	0	0	*82*
	*BR* 3	0	0	13	4	0	*17*
	*BR* 4	0	1	1	16	7	*25*
	*BR* 5	0	0	0	0	5	*5*
	Total	*54*	*83*	*15*	*21*	*13*	
			***κ*** = **0.821**		***IA*** *= 0.729*

**Table 3:** Cohen’s kappa and *IA* between US and ABVS in dichotomised BI-RADS classes (***BR*** in the table for brevity)

		US		
		*BR* 1-2	*BR* 3-4-5	Total
ABVS	*BR* 1-2	136	3	*139*
	*BR* 3-4-5	1	46	*47*
	Total	*137*	*49*	
	***κ*** **= 0.944**		***IA*** **= 0.836**	

In this reference study, previously diagnosed (e.g. by mammography and/or US with subsequent breast biopsy) breast cancers are staged with *magnetic resonance imaging* (MRI), in order to plan patient’s management. MRI usually detects additional findings with respect to the ones that prompted the examination [25]. New MRI findings can be characterised by the so-called second-look US, which, however, requires patient recalls. Since ABVS images can be stored and used at any time, [25] investigated whether US and ABVS agree at a reasonable extent in classifying MRI findings, in order to be used interchangeably as a second-look procedure in breast cancer staging. In particular, [25] used US and ABVS to classify 186 additional MRI findings in 131 women; they were classified independently and in blinded fashion, on the basis of both US and ABVS data according to the standardised 5-grade scale *Breast Imaging Reporting And Data System* (BI-RADS) lexicon [18]. Depending on the need of further clinical actions (e.g. additional biopsy), BI-RADS assignments were dichotomised into “*not significant findings*” (BI-RADS 1-2) vs. “*significant findings*” (BI-RADS 3-4-5). Dichotomisation is obtained in an obvious way; for example *n*_*D*_(1,1) of the dichotomised matrix is computed as ${\sum }_{i,j=1}^{2}n_{M}(i,j)$ of the multivalue matrix; *n*_*D*_(2,2) as ${\sum }_{i,j=3}^{5}n_{M}(i,j)$ and so on. The cancer detection rate observed in the referring study (i.e. the ratio between the number of cancers found on US or ABVS and the number of MRI findings proven to be malignant) was 83.8% for ABVS and 87.0% for US.

Finally, we investigate the relation between *IA* and *κ* by evaluating a few agreements of a few machine learning techniques on some data sets from the UCI Machine Learning Repository [19]: the Congressional Voting Records Data Set (DS0) [39], the Breast Cancer Wisconsin (Diagnostic) Data Set (DS1) [52], the Iris Data Set (DS2) [21], the Spambase Data Set (DS3) [27], the Tic-Tac-Toe Endgame Data Set (DS4) [3], and the Heart Disease Data Set (DS5) [28]. For each of these data sets, random forest, k-NN, stochastic gradient (SGD) and naïve Bayes models are trained by using 10-fold cross validation on the Orange Data Mining Toolbox [17]. All the data set entries are, then, labelled as either correctly (C) or wrongly (W) classified by each model and, for each pair of models, a comparison matrix is built: the first row/column of this matrix is devoted to the data set entries that are correctly classified by the first/second model in pair, while the misclassified entries are counted in the second row/column (e.g. see Table 5). These matrices enable us to compute both *IA* and *κ* for all the data sets and for all the pairs of ML models. To conclude the analysis, we fix an arbitrary ordering among all the pairs of models (in particular, random forest-kNN (FK), random forest-SGD (FS), random forest-naïve Bayes (FB), kNN-SGD (KS), kNN-naïve Bayes (KB), and SGD-naïve Bayes (SB) and we evaluate both Pearson’s correlation coefficient (*ρ*) [38] and Spearman’s rank correlation coefficient (*r*_*s*_) [48] between the ordered sequences of *IA*’s and *κ*’s to quantify how much a *IA*-*κ* switch may affect the relative agreement relations between the ML models.

## Results

Table 1a and b present two scenarios which have almost the same *κ*, i.e. about 0.5. However, from an intuitive point of view, the matrix associated with Table 1b seems to deserve a higher agreement among the two, because 99.34% of the subjects are classified in the same way by the two raters, while the percentage decreases to 73.4% in Table 1a. In these cases *IA* better matches the user expectations, since it equals 0.309 for Table 1a, while it is 0.651 for Table 1b.

Table 1c reports a highly symmetrically unbalanced (SU) matrix. Even though the overall probability of an agreed evaluation is high (0.991), *κ* is quite low, (0.228) while *IA*, whose value is 0.541, offers a more convincing result with respect to the common sense.

Tables 1d and e report two interesting scenarios: the former contains an asymmetrical unbalanced marginals (AU) matrix, while the latter, which was also discussed in [20, 43], is a case of highly symmetrically unbalanced marginals. They both deal with 50 samples, and 84% of them were classified in the same way by the two raters; however, while the evenly rated samples are uniformly distributed along the main diagonal in Table 1d, they are mostly gathered in position (1,1) in Table 1e. The elements in positions (2,1) and (1,2) remain unchanged in the two matrices. Intriguingly, even though the number of samples in the main diagonals of the two matrices is exactly the same, the linear scale proposed in [33] to rate *κ*—i.e. [0.0,0.2] (“*none to slight*”)^1^, [0.2,0.4) (“*fair*”), [0.4,0.6) (“*moderate*”), [0.6,0.8) (“*substantial*”), and [0.8,1.0] (“*almost perfect agreement*”)—classifies the scenario in Table 1d, where *κ* = 0.681, as a substantial agreement and that in Table 1e, where *κ* = 0.245, as a fair—and not even moderate—agreement. Of course, this scale cannot be directly applied to *IA*; however, the values of *IA* for Table 1d and e—i.e. 0.371 and 0.073, respectively—although different, do not appear to be so qualitatively dissimilar, since both are well below a value that it is reasonable to consider index for a substantial agreement.

A further comparison between Table 1c and d may also emphasise that there are cases (Table 1c) in which the percentage of evenly rated samples is extremely high (99%), but *κ* is rather low (*κ* = 0.228), and cases in which a smaller percentage corresponds to a significantly higher *κ*, such as in Table 1d (84% and *κ* = 0.681). Also for these scenarios, *IA* seems to out-perform *κ* and, consistently with common sense expectations, provides a higher value for matrix in Table 1c than for that in Table 1d.

Table 1f reports a scenario which is quite similar to the one presented in Table 1e. The values in the two matrices are almost identical, with the exception of 3 samples which are differently rated. This difference corresponds to changing only 6% of all the pairs of ratings (3%, if we decouple the evaluations of the two raters), but produces an increase in *κ* which is relevant with respect to the already discussed scale introduced in [33]; as a matter of fact *κ* rises from 0.228 (i.e. fair agreement) in Table 1e to 0.681 (i.e. substantial agreement) in Table 1f. Also the value of *IA* rises as *κ*, but it changes from 0.073 to 0.342, remaining around the one third of the scale maximum, that is well below a value for a substantial agreement.

Scenario 5 in Table 1e may raise some concerns about *IA* because, despite a high percentage of evenly rated samples, i.e. 42 over 50, *IA* is almost null, i.e. 0.073. However, this outcome is motivated by the fact that a large fraction of the rates in the second row (3/5) and a huge fraction of the rates in the second column (5/7) are misinterpreted by the two raters’ behaviour.

As for the agreement between US and ABVS in the clinical scenario of Tables 2 and 3 we can note that *κ* on the multivalue and on the dichotomised versions of ABVS and US was about *κ* ≈ 0.821 and *κ* ≈ 0.944, respectively. In both cases, the agreement was classified as “*almost perfect agreement*”. As for the *IA* index, it was about 0.729 for the multivalue and about 0.836 for dichotomised assessments.

We have to underline that while *IA* ranges in the interval [0,1], *κ* lies in [− 1,1]; thus, a direct comparison between these numerical values is not possible, also because only positive values of *κ* are considered in diagnostic practice; but, in any case, we can observe a fair behaviour of IA from an intuitive point of view.

**Fig. 2:**
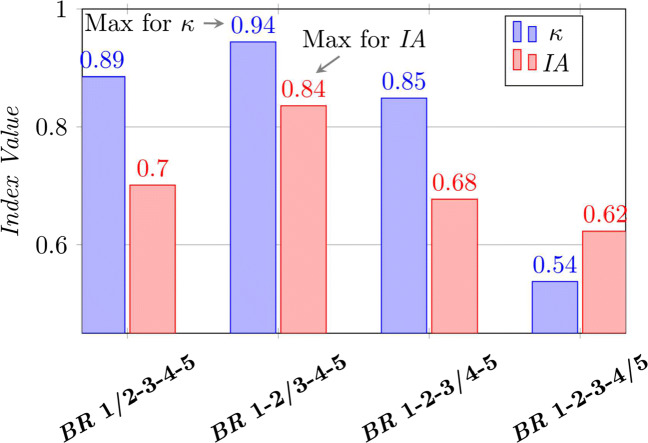
Choosing the best threshold in dichotomising a multivalue ordered-categorical ratings. The maximum agreement is obtained in correspondence with the standard dichotomisation 1-2/3-4-5 for *κ* and *IA*

As far as the dichotomisation threshold concerns, the reference partition 1-2/3-4-5 is validated by the highest agreement values for both *κ* and *IA* (see Fig. 2); so, from this point of view also, *IA* confirms its fair behaviour.

Finally, the machine learning scenario depicted at the end of Section 2.5 was considered and Table 4 was produced. Spearman’s rank correlation coefficients (*r*_*s*_) reported in the table highlight that *IA* and *κ* produce different ranking for all the data sets and proves that they are not strictly equivalent. However, Pearson’s correlation coefficient (*ρ*) approaches to 1 and, thus, *IA* and *κ* are significantly correlated for all the data sets but DS4. Therefore, in these cases, the ranking difference of the two indexes is due to swaps between pairs whose agreement measures are close and, from a qualitative point of view, the two indexes behave in the same way.

**Table 4:** A comparison between *IA* and *κ* on a Machine Learning domain

		FK	FS	FB	KS	KB	SB	*ρ*	*r*_*s*_
DS0	*IA*	0.28 (5)	0.42 (2)	0.30 (3)	0.28 (4)	0.56 (1)	0.19 (6)	0.98	0.77
	*κ*	0.46 (3)	0.63 (2)	0.43 (5)	0.44 (4)	0.72 (1)	0.31 (6)		
DS1	*IA*	0.54 (2)	0.33 (6)	0.71 (1)	0.36 (5)	0.53 (3)	0.39 (4)	0.92	0.83
	*κ*	0.73 (2)	0.56 (5)	0.77 (1)	0.58 (4)	0.63 (3)	0.54 (6)		
DS2	*IA*	0.63 (1)	0.52 (2)	0.22 (4)	0.47 (3)	0.17 (5)	0.14 (6)	0.98	0.94
	*κ*	0.79 (1)	0.64 (2)	0.37 (4)	0.57 (3)	0.30 (6)	0.35 (5)		
DS3	*IA*	0.14 (4)	0.33 (1)	0.28 (2)	0.08 (6)	0.10 (5)	0.19 (3)	0.93	0.94
	*κ*	0.21 (5)	0.51 (1)	0.41 (2)	0.20 (6)	0.28 (4)	0.41 (3)		
DS4	*IA*	0.11 (3)	0.25 (1)	0.18 (2)	0.04 (6)	0.11 (4)	0.04 (5)	0.61	0.60
	*κ*	0.15 (4)	0.29 (2)	0.17 (3)	0.08 (5)	0.36 (1)	0.03 (6)		
DS5	*IA*	0.05 (6)	0.28 (3)	0.43 (1)	0.06 (5)	0.06 (4)	0.34 (2)	0.99	0.77
	*κ*	0.23 (4)	0.55 (3)	0.67 (1)	0.21 (5)	0.21 (6)	0.62 (2)		

Six data sets from the UCI Machine Learning Repository [19] were considered: the Congressional Voting Records Data Set (DS0) [39], the Breast Cancer Wisconsin (Diagnostic) Data Set (DS1) [52], the Iris Data Set (DS2) [21], the Spambase Data Set (DS3) [27], the Tic-Tac-Toe Endgame Data Set (DS4) [3], and the Heart Disease Data Set (DS5) [28]. Each of the data sets were used to train random forest, k-nearest neighbours, stochastic gradient (SGD) and naïve Bayes models. Then the pairs of models random forest-kNN (FK), random forest-SGD (FS), random forest-naïve Bayes (FB), kNN-SGD (KS), kNN-naïve Bayes (KB), and SGD-naïve Bayes (SB) were compared according to their correct classifications of the data set entries and their *IA* and *κ* were evaluated. Finally, the Spearman’s rank correlation coefficient (*r*_*s*_) [48] between the sequences of *IA* s and *κ* s was computed. All the reported values were rounded up to the second decimal digit. The numbers inside round parentheses in the table represent the rank of the associated value among those on the same row

**Fig. 3:**
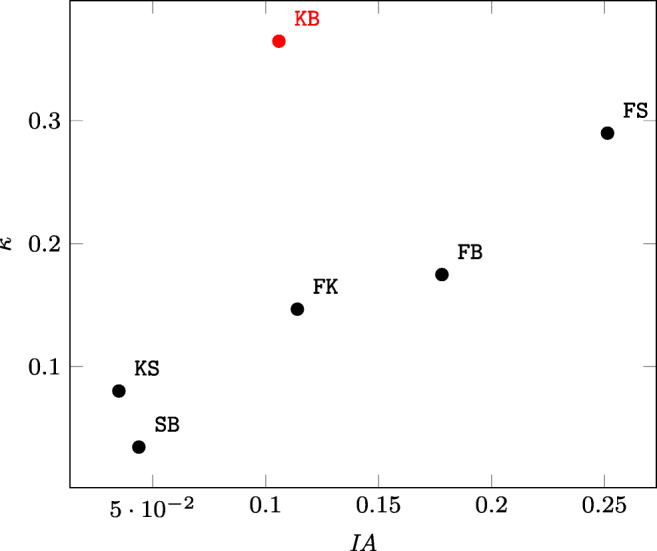
A scatter plot of the *IA*-*κ* values for the pairs of models random forest-kNN (FK), random forest-SGD (FS), random forest-naïve Bayes (FB), kNN-SGD (KS), kNN-naïve Bayes (KB), and SGD-naïve Bayes (SB) trained on the Tic-Tac-Toe data set. It is easy to see that the black points are strictly correlated, while the red one, corresponding to KB, falls apart from any reasonable model for the former

As far as the Tic-Tac-Toe data set (DS4) is concerned, the low *ρ* value is mainly due to the comparison between the kNN and the naïve Bayes models (KB) (see Fig. 3). So, in order to understand why KB is so special, we considered the random forest-stochastic gradient comparison (FS), which produced a higher *κ* with respect to KB, but a lower *IA*, and we tried to relate its agreement matrix and the one of KB itself (see Table 5a and b, respectively) to the corresponding index values. Because of the lower number of consistent classifications in Table 5a (704) with respect to the ones in Table 5b (913), common sense suggests that the latter should have an agreement higher than that of the former. However, while *IA* satisfies this intuition, *κ* does not and, once again, *IA* is to be more adherent to common sense than *κ*.

**Table 5:** Two agreement matrices relating the classifications performed by the pairs kNN and naïve Bayes models (KB) (Table 5a) and random forest and SGD models (FS) (Table 5b) on the Tic-Tac-Toe data set (DS4). The first rows/columns of these matrices count the correctly classified entries (C), while those misclassified (W) are packed in the second rows/columns

		Naïve Bayes				SGD	
		C	W			C	W
kNN	C	547	134	Random	C	903	6
	W	120	157	Forest	W	39	10
(a) The agreement matrix relating the classifications performed by kNN and naïve Bayes models (KB). Its *IA* and *κ* are 0.11 and 0.36, respectively				(b) The agreement matrix relating the classifications performed by random forest and SGD models (FS). Its *IA* and *κ* are 0.25 and 0.29, respectively			

## Discussion

Above results show the effectiveness of *IA* in the considered cases. They are analogous to those produced by *κ*; however, while the latter lacks a clear operative interpretation, the former has a formally defined and still intuitive meaning: it measures the quantity of normalised information exchanged between the two raters through the agreement channel.

Usually, *IA* is smaller than *κ*, and this is because *MI* features a steep front for *p*_*X*_(1) ∈ [0,0.4], and saturates when *p*_*X*_(1) ∈ [0.4,0.5]. This behaviour is typical for a measure of information, and is induced by the entropy-like feature of *MI*. Thus, tiny variations of *p* in the neighbourhood of 0 produce a huge change in *MI* and, as a consequence, *IA* seems to discriminate high quality agreements better than *κ*. Moreover, while *κ* is a function of only the main diagonal elements of the ratings matrix *R* and, thus, it exclusively evaluates agreement between raters, the elements laying outside the *R* main diagonal play a role in *IA* and, because of this, *IA* is a more complete measure of the relation between the two raters’ choice s. From this point of view, *IA* is a natural extension and completion of *κ*.

*IA* confirmed its fair behaviour—with respect to *κ*—in finding the best threshold for the dichotomisation of a multivalue ordered-categorical diagnostic scale.

In most of the considered machine learning scenarios, *IA* and *κ* exhibited similar qualitative behaviours. In the single case in which they significantly diverged, *IA* appeared to be more adherent to common sense than *κ*.

Digging for disadvantages of the proposed approach, we must emphasise that *IA* is more difficult to be calculated than *κ* because it involves logarithms and, due to the very same reason, whenever the agreement matrix contains some 0, *IA* cannot be computed. The former point is a minor issue and it can easily be overcome by using custom software. As far as the latter may be concerned, an extension of the proposed index for continuity seems to be sufficient to bypass the problem. This could be obtained, for instance, by replacing all the 0s in the agreement matrix with a new variable *𝜖* and, then, by computing *IA* on new matrix as *𝜖* tends to 0 from the right (e.g. as in $\lim _{x\rightarrow 0+} x \log x = 0$). Again, all these steps can easily be implemented in custom software.

## Conclusions

We have proposed an information theoretic model to evaluate the agreement between two raters; this has been made by gauging the information flow between the rater $\mathfrak {X}$, the patient condition $\mathfrak {D}$, and the second rater $\mathfrak {Y}$. This is done by means of the agreement channel, which consists of the concatenation of the two diagnostic channels $\mathfrak {X}$-to-$\mathfrak {D}$ and $\mathfrak {D}$-to-$\mathfrak {Y}$. The more information is exchanged between $\mathfrak {X}$ and $\mathfrak {Y}$, the more the agreement between the two readers. This approach uniformly handles both the dichotomous and the multivalue ordered-categorical case. The strong foundation of *IT* gives an added value to *IA*, when compared with Cohen’s kappa: it expresses a clear operative interpretation of the agreement, based on an objective measure of the normalised information exchanged between the raters $\mathfrak {X}$ and $\mathfrak {Y}$ through the agreement channel. The mathematical structure of *IA* and the fact that it takes into account both the agreement and the disagreement contributions in quantifying the relation between raters’ evaluations, shows that it is a natural extension and completion of *κ*.

We have shown that *IA* corrects some flaws of *κ*; moreover, by using *IA* we are able to separate the contribution of *P*_*X*_, that’s tied with the prevalence of disease, from that of the matrix *Γ*_*q*_, representing the agreement channel, which constitutes the essential nucleus of agreement; this is an important conceptual step. The results of the comparison between *κ* and *IA* in the context of machine learning and in a real case-of-study, connected to breast cancer imaging, validates the use of *IA* in a real diagnostic scenario, showing, in this specific case, the practical fair behaviour of *IA* with respect to *κ*, both for the classification issue and for the best threshold when dichotomising a multivalue BI-RADS scale.

As for the future work, in order to spread the proposed method and provide researchers with tools to easily adopt it, we plan to develop a software library and a website for the evaluation of *IA* for both dichotomous and multivalue ordered-categorical *q*-levels scales.

## Footnotes

1 Negative values for *κ* are usually interpreted as “no agreement” and flattened to 0 (e.g. see [33]).
